# Combining spironolactone to antiretroviral therapy accelerates HIV decay in humanized mice

**DOI:** 10.1080/22221751.2025.2589549

**Published:** 2025-11-30

**Authors:** Lijun Ling, Luisa P. Mori, Andrew Soper, Wenbo Yao, Andrew T. McAuley, Ana R. Leda, Rae Ann Spagnuolo, Nurjahan Begum, Martina Kovarova, Angela Wahl, Michael D. Cameron, Susana T. Valente, J. Victor Garcia

**Affiliations:** a Department of Microbiology, University of Alabama at Birmingham, Birmingham, AL, USA; b International Center for the Advancement of Translational Science, University of North Carolina at Chapel Hill, Chapel Hill, NC, USA; c Division of Infectious Diseases, Department of Medicine, University of North Carolina at Chapel Hill, Chapel Hill, NC, USA; d Center for AIDS Research, University of North Carolina at Chapel Hill, Chapel Hill, NC, USA; e The Skaggs Graduate School of Chemical and Biological Sciences, The Scripps Research Institute, Jupiter, FL, USA; f Department of Immunology and Microbiology, The Herbert Wertheim UF Scripps Institute for Biomedical Innovation & Technology, Jupiter, FL, USA; g Department of Molecular Medicine, The Herbert Wertheim UF Scripps Institute for Biomedical Innovation & Technology, Jupiter, FL, USA

**Keywords:** Spironolactone, HIV-1 transcription, latency-promoting agents, humanized mouse model, Chronic inflammation

## Abstract

Spironolactone (SP), a clinically used aldosterone antagonist, has been explored as an anti-HIV agent in models of HIV-1 latency for inducing transcriptional silencing of the viral reservoir*.* SP promotes the degradation of xeroderma pigmentosum group B (XPB) protein, a crucial component of transcription factor II H (TFIIH) required for RNA polymerase II transcriptional initiation. This study evaluated the impact of a long-acting formulation of SP on HIV replication within the context of antiretroviral therapy (ART) in the humanized mice model of HIV infection. The findings demonstrate that adding SP to ART accelerates viral decline and reduces expression of inflammation-related genes in human immune cells – genes often upregulated in chronic viral infections. Although SP treatment did not alter levels of cell-associated viral DNA, it led to a significant 4.4-fold decrease in systemic cell-associated viral RNA. This supports the role of XPB in HIV transcriptional regulation and advocates for incorporating transcriptional inhibitors like SP into primary HIV therapy. Additionally, SP treatment diminished markers of immune activation and inflammation, critical factors contributing to morbidity and mortality in individuals with chronic HIV infection. These results highlight SP’s potential to enhance HIV treatment by mitigating key aspects of viral persistence and associated immune challenges.

## Introduction

Current antiretroviral therapy (ART) is highly effective in controlling HIV-1 replication, but it is not curative. When ART is interrupted, viral replication inevitably rebounds from a reservoir of latently infected cells. These latently infected CD4 ^+^ T cells are particularly challenging to detect and eradicate, as they do not express viral antigens, making them indistinguishable from uninfected cells. A functional cure for HIV-1, also referred to as remission, can be defined as long-term, sustained control of HIV-1 replication in the absence of ART, despite the presence of detectable integrated proviruses. To achieve a remission the viral reservoir must be rendered resistant to reactivation, and the proof of principle for this approach was demonstrated using the Tat inhibitor didehydro-Cortistatin A (dCA) [1]. dCA functions by blocking the activity of Tat, the key regulator of HIV-1 transcription, and drives the viral promoter into deep transcriptional inhibition or “deep latency” over time, in both *in vitro* and *in vivo* studies[2–5]. Although dCA holds great potential, the high cost of large-scale production has slowed progress towards clinical studies[6]. Additional transcriptional inhibitors are thus needed as part of the antiviral ammunition against HIV.

Spironolactone (SP) has been approved by the FDA for the treatment of hypertension, congestive heart failure, and other edematous conditions mainly through aldosterone antagonism[7,8]. An off-target effect of SP is the rapid induction of proteolytic degradation of the xeroderma pigmentosum group B (XPB) protein, a critical subunit of the transcription factor TFIIH, and previously shown to limit NF-κB and AP-1 signalling and reduce inflammatory signalling in human pulmonary artery endothelial cells[9]. Studies in cell line models and primary CD4 ^+^ T cells demonstrated that SP treatment significantly reduces RNAPII occupancy at the HIV-1 promoter, surpassing the effects of ART alone, effectively inhibiting HIV-1 transcription and preventing viral reactivation with latency reversing agents (LRAs)[10,11]. Additionally, maintaining SP alone after inducing deep latency with ART and SP is enough to keep HIV-1 deeply silenced. However, removing all drugs leads to an immediate viral rebound as the XPB protein restores equilibrium.

Here, we explored the impact of adding SP to ART in first-line treatment against HIV-1 infection in humanized mice. Long-acting SP pellets were implanted subcutaneously at the time of ART initiation in HIV-infected humanized mice, ensuring sustained release of SP. We demonstrate that SP accelerates the decay of HIV-1 in plasma viremia, outperforming ART alone, without impacting human immune cells in peripheral blood and tissues. As expected from a transcriptional inhibitor, SP significantly reduced cell-associated viral RNA levels, but not cell-associated viral DNA levels in tissues of humanized mice. We also observed a significant downregulation of inflammation-related gene expression. Our findings collectively indicate that SP treatment not only accelerates silencing of viral RNA expression *in vivo* HIV-1 reservoirs, but also mitigates chronic immune activation, a hallmark of HIV infection.

## Results

### Sustained release of SP in mice

SP has a relatively short plasma half-life of approximately 0.738 h in mice[12]. To achieve long-term drug release, subcutaneous long-acting SP pellets (200 mg/pellet/mouse) were implanted into the backs of adult NOD.Cg-*Prkdc^scid^Il2rg^tm1Wjl^*/SzJ (NSG; The Jackson Laboratory) mice (Figure S1A). The implant was well tolerated and SP achieved sustained release in mice, with mean (±SEM) plasma concentrations between day 1 and day 35 of 1.17 ± 0.2 µM (Figure S1B). Throughout the treatment period, mouse body weights, overall behaviour, fur coat, and healing at the site of pellet implantation were monitored and no overt toxicity or significant changes in weight were detected (Figure S1C). Therefore, SP was well-tolerated and long-term release successfully achieved.

### Combining SP to ART accelerates the decay of plasma HIV-1 in humanized mice

**Figure 1.**
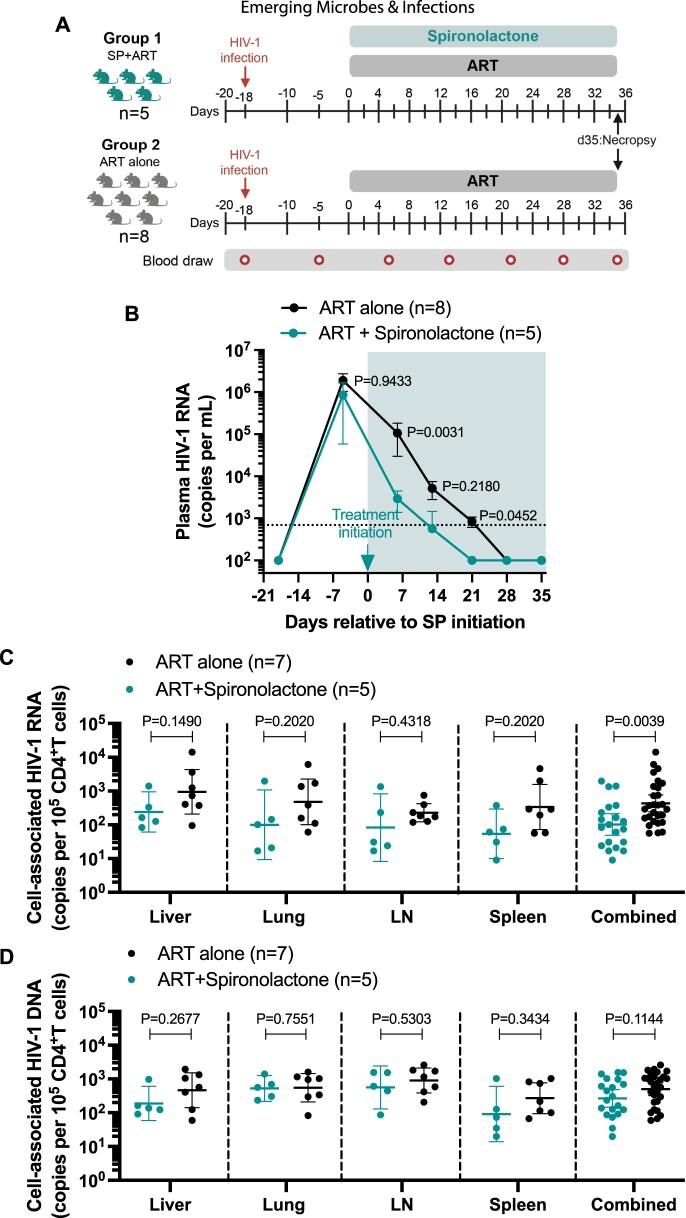
Spironolactone accelerates HIV-1 decay in plasma and decreases the levels of cell-associated HIV RNA but not DNA in tissues of HIV-infected mice at necropsy during ART suppression. (A) Experimental design. Humanized mice were intravenously exposed to HIV-1 on day −18. SP and ART were initiated on day 0. ART was administered via chow diet containing emtricitabine, tenofovir disoproxil fumarate and raltegravir (Research Diets). Animals were treated with either SP + ART (*n* = 5, turquoise) or ART alone (*n* = 8, black). Red arrow indicates infection with 30,000 _TCIU50_ HIV-1^JR-CSF^. Peripheral blood samples were collected on days −18, 6, 13, 21, 28 and 35 (small red circles). Mice were euthanized on day 35 and necropsy was performed. (B) Mean plasma HIV viral load of SP plus ART-treated animals (*n* = 5, turquoise) and ART alone-treated animals (*n* = 8, black). The limit of detection (693 copies/ml) is shown with a dashed line. Turquoise arrow indicates the initiation of SP. The shaded area shows the period of ART treatment. (C) Copies of cell-associated HIV RNA per 10^5^ CD4^+^ T cells in tissues of HIV-infected mice treated with SP plus ART (*n* = 5, red) or ART alone (*n* = 7, black). (D) Copies of cell-associated DNA per 10^5^ CD4^+^ T cells in tissues of HIV-infected mice treated with SP plus ART (*n* = 5, turquoise) or ART alone (*n* = 7, black). Data are presented as geometric mean with 95% confidence interval (CI). Statistical analyses were performed using unpaired two-sided Mann–Whitney U-tests. LN: lymph nodes. Combined: all individual tissues from all animals with the same group are plotted together. SP: spironolactone.

We evaluated the activity of SP combined with ART on inhibiting HIV transcription *in vivo* as part of first-line therapy (Figure 1A). We generated humanized mice (Table S1) and infected each animal intravenously (i.v.) with 3 × 10^4^ tissue culture infectious units (TCIU) of HIV-1_JR-CSF_. Starting day 18 post-infection, we initiated long-acting SP (LA-SP, 200 mg/pellet/mouse) and an ART-enriched chow diet (emtricitabine, tenofovir disoproxil fumarate and raltegravir, as previously described[13–16]). We monitored viral loads longitudinally in HIV-infected mice treated with SP plus ART (*n* = 5) or ART alone (*n* = 8) (Figure 1B). Significantly lower plasma viral loads were observed in the SP plus ART group compared to the ART alone group on day 7 (*p* = 0.0031) and day 21 (*p* = 0.0452) after SP initiation (Figure 1B). By day 21, all animals in the SP plus ART group had undetectable plasma viral loads, whereas only 3 out of 8 in the ART alone group reached this level. These findings collectively suggest that SP can accelerate HIV-1 suppression effectively when combined with ART *in vivo*.

### SP treatment does not alter humanization in peripheral blood or tissues of humanized mice

We longitudinally studied the potential cytotoxic effects of SP on human immune cells and T cell activation in HIV-infected humanized mice treated with ART alone or combined with SP. No significant differences were observed in the number of human CD45 + cells, or the frequency of CD3+, CD4+, or CD8+ T cells between the SP plus ART group and the ART-only group after starting SP treatment (Figure S2A-D). Similarly, levels of CD4 + and CD8+ T cell activation (CD38+/HLA-DR+) were consistent in the peripheral blood and tissues of both groups (Figure S3A-D). Further analysis showed no significant differences in human immune cell counts or percentages across various tissues between the two groups (Figure S4A-D). Overall, our findings indicate that SP treatment does not affect immune cell levels or T cell activation in the peripheral blood or tissues of HIV-infected, ART-treated humanized mice, aligning with expectations given the drug's FDA-approved status and tolerability in humans.

### SP treatment reduces systemic levels of cell-associated HIV RNA, but not HIV DNA, in HIV-infected, ART-treated humanized mice at necropsy

To explore the effects of SP on HIV-1 RNA levels in tissues, total RNA was extracted from mononuclear cells (MNCs). We observed a decrease in cell-associated viral RNA in the liver (7-fold), lung (3.1-fold), and spleen (9.7-fold) in the SP plus ART group compared to the ART alone group (Figure 1C). When pooling samples from all tissues, there was a significant 4.4-fold reduction in viral RNA levels in the SP plus ART group compared to those treated with ART alone (*p* = 0.0039) (Figure 1C). To account for inter-animal variability, we reanalyzed the data by pooling tissue measurements to generate a single composite value per animal. Using these per-mouse averaged values, we again found ∼4.4-fold lower mean RNA levels in the SP plus ART group compared to ART alone, although this difference did not reach statistical significance (*p* = 0.1061). This trend is consistent with the tissue-level analysis and suggests a transcriptional suppressive effect of SP that warrants confirmation in larger cohorts (Figure S5). An examination of cell-associated HIV DNA levels in these tissues showed no notable differences between the treatment groups (Figure 1D). To further account for inter-animal variability and evaluate transcriptional activity relative to reservoir size, we analyzed RNA/DNA ratios. Although differences did not reach statistical significance, consistent trends toward lower RNA/DNA ratios were observed in the SP plus ART group across multiple tissues: 45.8% reduction in the liver, 71.2% in the lung, and 64.1% in the spleen. When pooling data from all tissues, the ART + SP group showed an overall 55.2% reduction in RNA/DNA ratio compared to ART alone (Figure S6). The consistent DNA levels across both groups, together with the reductions in RNA/DNA ratios, suggest that the observed decrease in RNA levels is due to the suppression of integrated viral gene expression rather than the eradication of ongoing infections. These findings indicate that, in the context of acute 18-day infections, SP treatment can reduce cell-associated viral RNA levels without affecting viral DNA loads in tissues, reflecting suppression of viral transcription.

### SP treatment decreases the expression of inflammatory genes in the tissues of HIV-infected ART-treated animals

**Figure 2.**
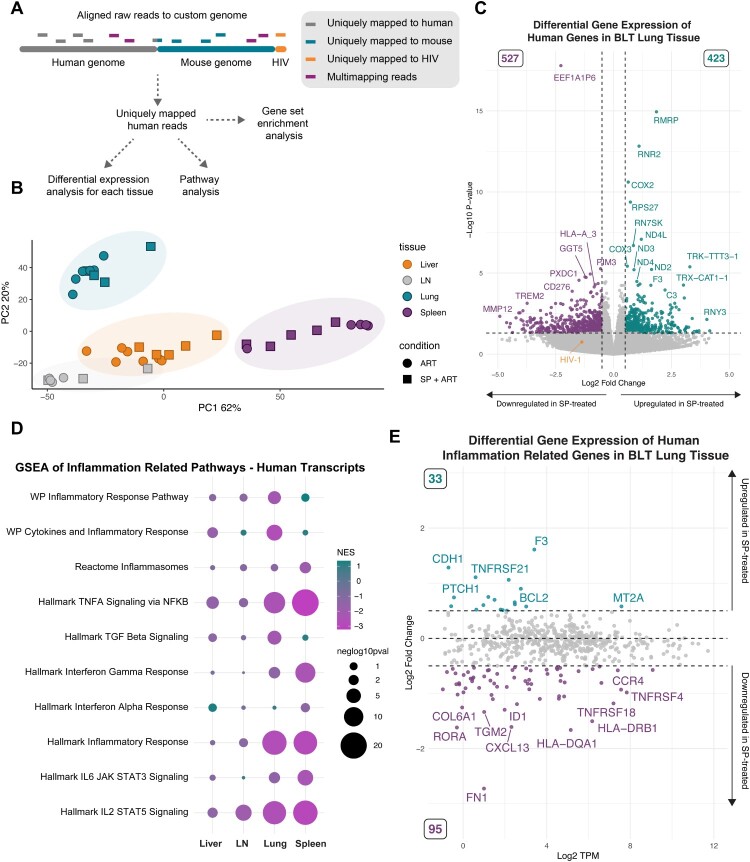
Treatment with SP decreases the expression of inflammation related genes in tissues of HIV-infected humanized mice treated with ART. (A) An outline of the RNA-seq analysis workflow. RNA isolated from mononuclear cells (MNCs) from indicated tissues of humanized mice treated with ART or ART + SP was subjected to total RNA-seq analysis. RNA-seq reads from lung samples were aligned to a custom human-mouse-HIV-1 genome and reads uniquely mapping to the human genome were analyzed individually. (B) Principal component analysis (PCA) of gene expression (using all uniquely mapped reads to the human, mouse or HIV-1 genomes) in different tissues of BLT mice treated with ART alone (circles) of ART + SP (squares). Each point represents a sample, the colour represents type of tissue, and shape represents treatment group. (C) Volcano plot of differential expression analysis of RNA expression in lung samples isolated from BLT mice treated with SP + ART versus ART alone, using only reads aligning uniquely to the human genome. Purple: downregulated with SP, Teal: upregulated with SP. Horizontal dashed line represents a p.value of 0.05. Vertical dashed lines represent a log2FC of −0.5 and 0.5. (D) Gene Set Enrichment Analysis of inflammation related human gene sets downloaded from MSigDB. (E) Differential gene expression of inflammation related genes in the gene sets in (D) in SP + ART versus ART alone treated animals, using only reads uniquely mapping to the human genome in the lung. Dashed lines represent a log2FC of −0.5 and 0.5. Inflammation related genes which were significantly differentially expressed upon SP treatment are highlighted in teal (Up) and purple (Down), [log2FC] > 0.5 and pvalue <0.05.

To study the genome-wide transcriptional effects of SP treatment in HIV-infected humanized mice, we conducted Total-RNA-seq analysis on the RNA extracted from MNCs from the liver, lung, spleen, and lymph nodes. Differential gene expression analysis was performed using reads mapping uniquely to the human or HIV-1 genome (Figure 2A). As expected, samples clearly separated based on tissue type, and also distinguished by treatment condition (Figure 2B). Analysis revealed approximately 300–900 genes with altered expression in each tissue due to SP treatment (Figure 2C and S7A-C). The RNA-seq study was conducted on day 35 of treatment when all mice had undetectable plasma HIV-1 RNA levels and low tissue levels by RT-qPCR (Figure 1B-C), thus HIV-1 RNA transcript levels in each tissue were very low (<1 transcript per million (TPM)). Nevertheless, a reduction in HIV-1 transcripts was observed in lung MNCs (log2 fold change = −1.4, *p*-value = 0.18) (Figure 2C, orange point). We then conducted pathway analysis of genes significantly upregulated upon SP treatment, pooled across all tissues, and found an enrichment of gene pathways related to translation, DNA repair and the cell cycle (Figure S7D, bold text). Among downregulated genes, significant enrichment was seen in genes related to extracellular matrix organization, cell signalling, and some immune signalling pathways (Figure S7E, bold text). Given the established roles of both HIV-1 replication and SP treatment in inflammatory processes[9,17–20], we focused our analysis on the effects of SP on inflammation-related genes in HIV-1-infected humanized mice. We performed gene set enrichment analysis (GSEA) on the differential gene expression from each tissue separately.

While tissue-specific differences existed, there was a significant enrichment of inflammation-related pathways among genes downregulated by SP treatment (Figure 2D). We curated a list of 780 unique inflammation-related genes from these gene sets (see Methods for details). Generally, approximately twice as many inflammation-related genes were downregulated than upregulated in liver, lung, and spleen samples from SP-treated mice (log2FC < −0.5, *p*-value < 0.05) (Figure 2E, S7F-G). Conversely, in the lymph nodes, there was a trend toward increased expression of inflammation-related genes (Figure S7H). Notably, no suppression of HIV-1 RNA was observed by RT-qPCR (Figure 1C) or RNA-seq (Figure S7C) in the lymph nodes with SP treatment. The observed repression of inflammation-related gene expression in SP-treated cells may be linked to the suppression of HIV-1 RNA expression.

## Discussion

In this study, we evaluated the potential of SP, a clinically approved drug, to inhibit HIV transcription alongside first-line ART in humanized mice. We sought to determine whether adding a transcriptional inhibitor could enhance HIV-1 suppression and reduce residual transcriptional activity during ART. While no HIV-1-specific transcriptional inhibitors are clinically available, several compounds have been investigated as anti-HIV agents[21]. Theoretically, inhibiting HIV-1 transcription during effective ART could prevent viral reactivation from latency and suppress ongoing viral transcription, which is still detected during suppressive ART[22–26]. Notably, persistent low-level viremia, viral blips (brief detectable viremia periods), and cell-associated viral RNA are common in many ART-suppressed PLWH[26] and are linked with a slower viral reservoir decline[27], immune activation, disease progression, and accelerated time to viral rebound following treatment interruption[22–25].

Our results showed that SP, when administered in a sustained-release form combined with ART, achieved high nanomolar serum concentrations, demonstrated excellent safety and tolerability, and notably, led to a 2-log reduction in plasma viral load within a week of treatment initiation. Variations in spironolactone levels over time are expected for matrix-based slow-release systems, where drug release gradually slows due to dissolution, erosion, and diffusion without controlled mechanisms[28]. Adding SP to ART also accelerated viral suppression to below detectable levels by one week, compared to ART alone. Additionally, we observed a significant reduction in cell-associated viral RNA in SP treated tissues, although proviral DNA content remained similar between treatment groups. Analysis of RNA/DNA ratios further supported this trend, showing consistent reductions across tissues, which suggests that SP suppresses integrated viral gene expression. A complementary analysis using per-mouse averaged tissue values also showed a similar trend of reduced RNA levels in the SP plus ART group, though this difference did not reach statistical significance, highlighting the need for larger cohorts to confirm this effect. One might expect a reduction in cell-associated DNA with accelerated HIV-1 suppression via SP, but this was possibly influenced by the treatment beginning 18 days post-infection when a stable viral reservoir had already been established. Early intervention with SP, combined with ART, might show reduced levels of integrated viral DNA and assist in reducing the viral reservoir.

Considering that ongoing viral transcription during suppressive ART is associated with chronic inflammation, we analyzed transcriptomic changes using total RNA-Seq of RNA isolated from MNCs in tissues. SP was found to reduce the expression of inflammatory genes, especially those in the TNF-α signalling pathway via NF-κB and the IL2-STAT5 signalling pathways.

Although HIV RNA transcript levels were low at the time of the analysis, the lung, which exhibited the greatest reduction in HIV-1 transcripts (log2FC = −1.31; *p* = 0.18), also showed the largest decrease in inflammation-related gene expression. It is conceivable that SP's acceleration of viral suppression decreases tissue inflammation. Moreover, SP's known anti-inflammatory properties may also contribute to these effects, as previous studies reported reduced inflammation-related biomarkers with SP therapy in human pulmonary artery endothelial cells and in patients with pulmonary arterial hypertension[9].

While promising, our study has limitations. First, the relatively small cohort size (*n* = 5 in the SP plus ART group and *n* = 7 in the ART alone group), as well as the imbalance between treatment arms, likely reduced statistical power and contributed to variability in the results, particularly for the per-mouse analyses where only trends were observed. The humanized mouse model, though reflective of key HIV infection aspects, does not fully recapitulate the complexity of latent reservoirs in long-term infections. Although higher doses of SP were well-tolerated, the long-term effects on immune homeostasis warrant further investigation. For context, therapeutic doses of SP in humans (25–200 mg daily) yield peak plasma concentrations (Cmax) of up to to 0.44 µM at 200 mg daily [29], with other studies reporting Cmax values of 0.19 µM to 0.33 µM at 100–200 mg daily [30]. In humans, SP peaks in plasma at about 1.5 h post-dose, with a half-life of approximately 6.5 h. In our mice, mean (±SEM) plasma concentrations between days 1 and 35 were 1.17 ± 0.2 µM, generally exceeding typical human levels, and remained stable without noticeable peaks, due to steady drug release from subcutaneous pellets. However, species differences, particularly mice’s higher metabolic rates and faster drug clearance, limit direct comparison of plasma levels and dosing. Future research should assess the durability of SP-induced transcriptional silencing with extended treatment and explore whether combining SP with other latency-promoting agents can further suppress HIV-1. Ongoing studies are exploring the benefits of combining SP with other inhibitors targeting additional transcription steps.

This study is the first to demonstrate that adding SP to first-line ART accelerates viral suppression and reduces residual viral RNA in tissues–a novel outcome not achieved by adding a fourth antiretroviral to current ART regimens[31–34]. Thus, SP holds substantial promise as a co-treatment with ART. These findings suggest a potential dual-purpose role for SP in enhancing ART by accelerating viral decay and reducing inflammation, which addresses both residual viremia and chronic inflammation seen during ART. Further preclinical and clinical studies are required to confirm these results and evaluate the practicality of integrating SP into HIV treatment strategies.

## Materials and methods

### Ethics statement

Animal experiments were carried according to protocols approved by the Institutional Use and Care Committee of the University of North Carolina at Chapel Hill and in accordance with the NIH Guide for the Care and Use of Laboratory Animals.

### *In vivo* SP administration

Long-acting Spironolactone (LA-SP) pellets (200 mg, Innovative Research of America) were subcutaneously implanted into the back of shaved, anaesthetized NSG mice. Plasma samples were collected post-implantantion to measure SP concentrations.. Sodium fluoride was used to inhibit SP degradation by plasma esterases[35].

### Tandem mass spectrometry coupled to liquid chromatography (LC/MS/MS)

The concentration of SP was determined by LC-MS/MS. A 5 µL smaple of mouse plasma was directly loaded onto a 96-well Millipore Multiscreen Solvinter 0.45-micron low-binding PTFE hydrophilic filter plate containing 75 µL 90/10 acetonitrile/water with Carbamazepine as an internal standard to extract SP and precipitate protein. The plates were agitated on ice for ten minutes prior to centrifugation into a collection plate. The filtrate was analyzed on a Sciex 5500 operating in positive ion mode. SP was detected using a parent ion of 417 and fragment ion of 341, with the decluster potential set to 150 and collision energy at 27.

### Generation of humanized mice

Bone marrow liver and thymus (BLT) humanized mice were generated as previously described[13–16].

### HIV production and infection of humanized mice

Stocks of HIV-1_JR-CSF_ were produced and titrated as previsouly described[13]. Humanized mice were intravenously (via tail vein) injected with 3 × 10^4^ tissue culture infectious units (TCIU) HIV-1_JR-CSF_.

### ART administration and treatment of SP in humanized mice

ART was delivered via irradiated Teklad chow diet containing emtricitabine, tenofovir disoproxil fumarate and raltegravir (Research Diets) as previously described[13,15,16].

### Mononuclear cell isolation

MNCs were isolated from spleen, lymph nodes, bone marrow, liver, lung and human thymic organoid as described previously[15,16,36].

### Plasma viral load and quantification of cell-associated HIV-1 RNA and DNA in humanized mice

Plasma HIV viral loads were longitudinally monitored throughout infection. HIV RNA was detected using one-step reverse-transcriptase qPCR with a TaqMan RNA-to-Ct 1-Step kit, as previously described[13–16].

qPCR was performed to simultaneously detect the copy number of cell-associated viral DNA and human globin DNA in the peripheral blood and tissue cells of humanized mice, as previously described[36,37].

### Flow cytometric analysis

A multicolour flow cytometry panel was utilized to detect human immune cells in peripheral blood and tissue samples from humanized mice (Figure S6). The panel included antibodies such as anti-human CD45-V500, CD3-APC-R700, and CD19-PE-Cy7 to identify general immune cells, T cells, and B cells, respectively. CD4 + and CD8+ T cells were tagged with anti-human CD4-APC-H7 and CD8-FITC. T cell activation was assessed using co-expression markers CD38 (anti-human CD38-APC) and HLA-DR (anti-human HLA-DR-PerCP). The staining panel also included antibody isotypes like mouse anti-human IgG1k in various fluorochromes and mouse anti-human IgG2ak-PerCP. Analysis was performed using a BD LSRFortessa instrument and data were processed with BD FACSDiva (version 6.1.3) and FlowJo (version 10.6.2) software.

### Total RNA sequencing

Total RNA Sequencing: Cellular RNA (CA-RNA) was extracted and quantified using a Qubit 2.0 Fluorometer (Invitrogen, Carlsbad, CA). Quality was assessed on an Agilent 2100 Bioanalyzer Nano chip for liver, lung, and spleen samples, and an Agilent 4200 TapeStation for lymph node samples. We depleted ribosomal RNA using the NEBNext rRNA depletion module and prepared the libraries following the NEBNext Ultra II Directional RNA kit protocols. RNA samples were fragmented, reverse transcribed into first-strand cDNA, and the second strand synthesized with dUTP replacing dTTP for directional sequencing assurance. Adaptors were ligated to cDNA, and the second strand was degraded using USER enzyme to preserve the first strand. Libraries, normalized to 4nM and pooled, were sequenced on a NextSeq 2000 P3 flow cell at 750 pM using 2 × 50 bp paired-end chemistry, generating an average of 30 million reads per sample.

### Total RNA sequencing analysis

Read quality was assessed using FastQC (Braham Bioinformatics) and MultiQC[38]. A custom genome was created by merging the human genome (GRCh38.p14), mouse genome (GRCm39), and HIV-1 genome (NC_001802.1). Raw FASTQ files were aligned to this genome using STAR[39]. Only reads uniquely mapping to human, mouse, or HIV-1 transcripts were used for further analysis. Aligned transcriptomes were assigned to genes with featureCounts[40], and transcripts per million (TPMs) were determined by normalizing counts to gene length and library size. Differential gene expression analyzed with DESeq2[41], and plots were cretaed in R using ggplot2[42]. Gene pathway analysis was performed on a ranked gene set from the differential expression analysis (ART + SP versus ART) using ReactomePA, focusing on uniquely mapped human reads[43,44].

### Analysis of inflammation related gene expression

A curated list of inflammation-related genes was generated by searching for keywords “inflamm”, “interferon” and “signaling” in gene sets from the Human Molecular Signatures Database (MSigDB) collections, including “curated gene sets” (*n* = 7233), “hallmark gene sets” (*n* = 50), and “TFT: transcription factor targets” (*n* = 1115). The fgsea package was used to conduct gene set enrichment analysis[45–48]. A list of inflammation-related genes was generated by merging all unique genes from the selected sets.

### Statistical analyses

Graphs and analysis utilized GraphPad Prism (version 10.4.1). Data are presented as geometric mean with 95% confidence interval (CI) or mean ± SEM, with *p* < 0.05 denotating statistically significant. Differences between the SP plus ART-treated group and the ART alone-treated group were evaluated using unpaired two-sided Mann–Whitney U-tests. Within the same group, differences were assessed using the Wilcoxon Signed Rank test.

## Supplementary Material

Ling short report Supp figs.pdf

## Data Availability

All data are contained within the manuscript or its supporting documents. RNA-sequencing data will be deposited to Gene Expression Omnibus (GEO), and can be accessed with accession number GSE310371.
