# Supplementary figures and images for: Combining spironolactone to antiretroviral therapy accelerates HIV decay in humanized mice
Source: Emerg Microbes Infect. 2025 Nov 30;14(1):2589549. doi: 10.1080/22221751.2025.2589549 (PMC12667289; doi:10.1080/22221751.2025.2589549)

Figure S1

A

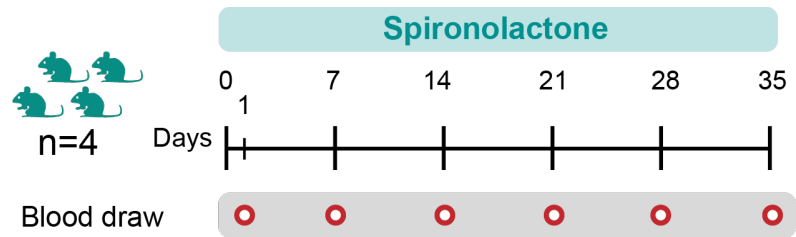

B

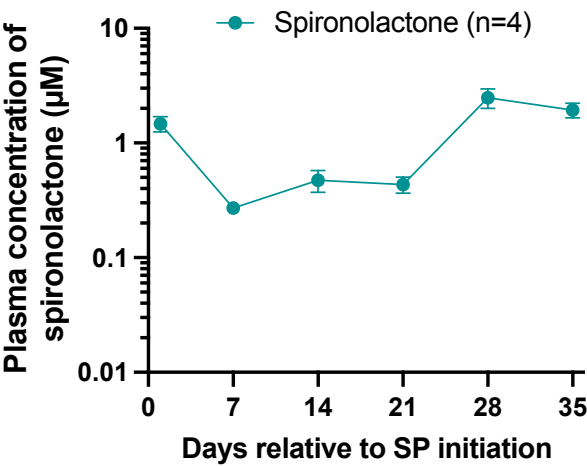

C

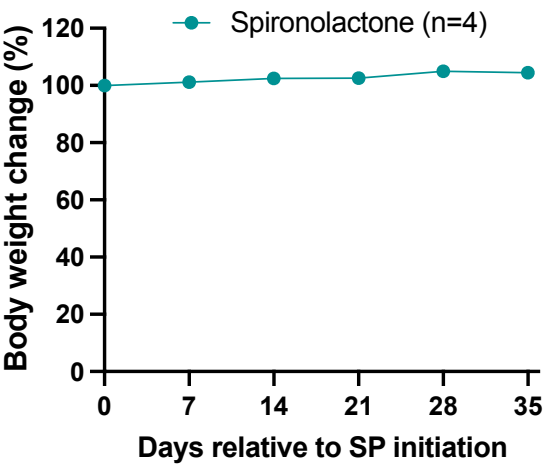

Figure S2

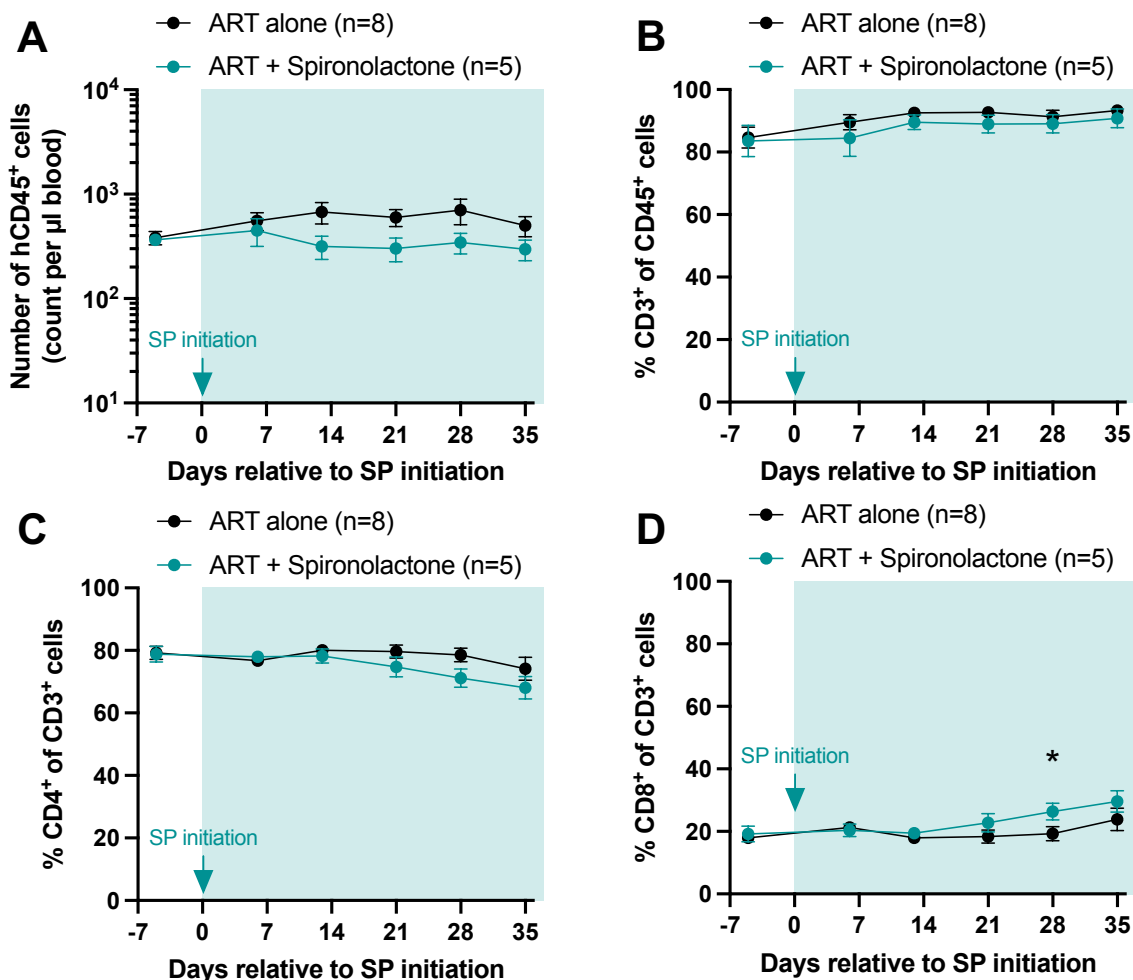

Figure S3

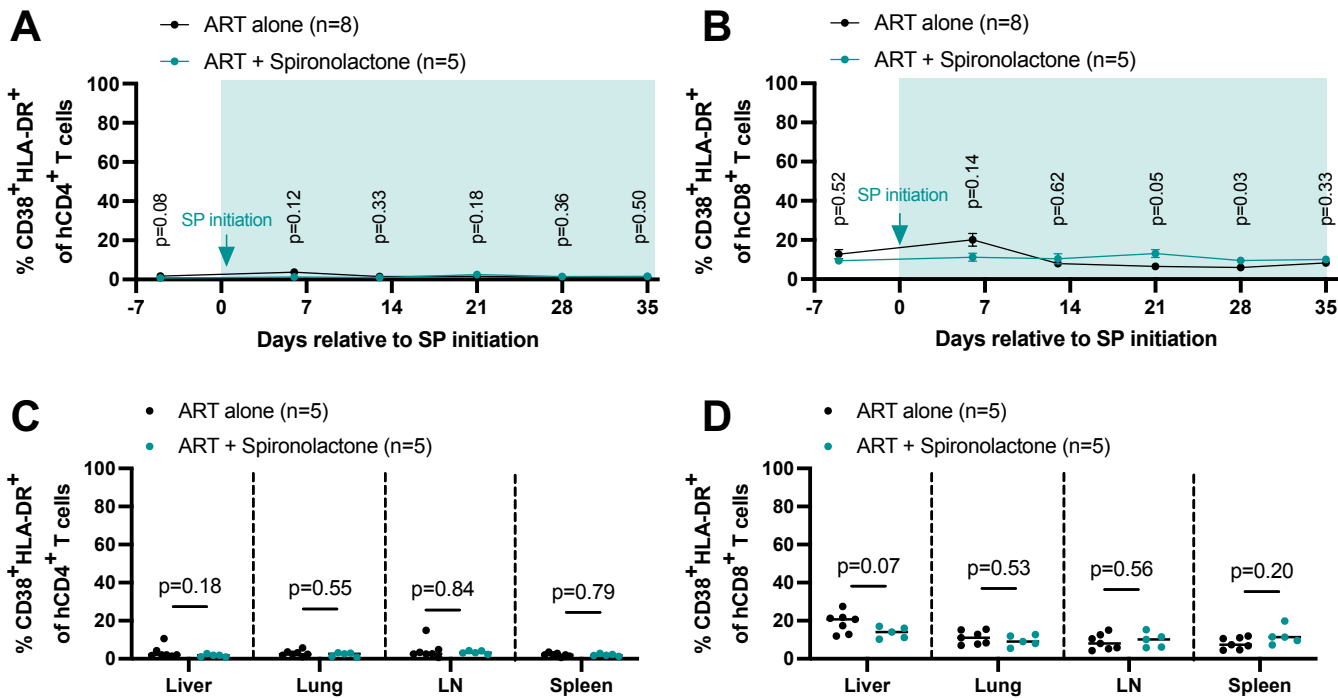

Figure S4

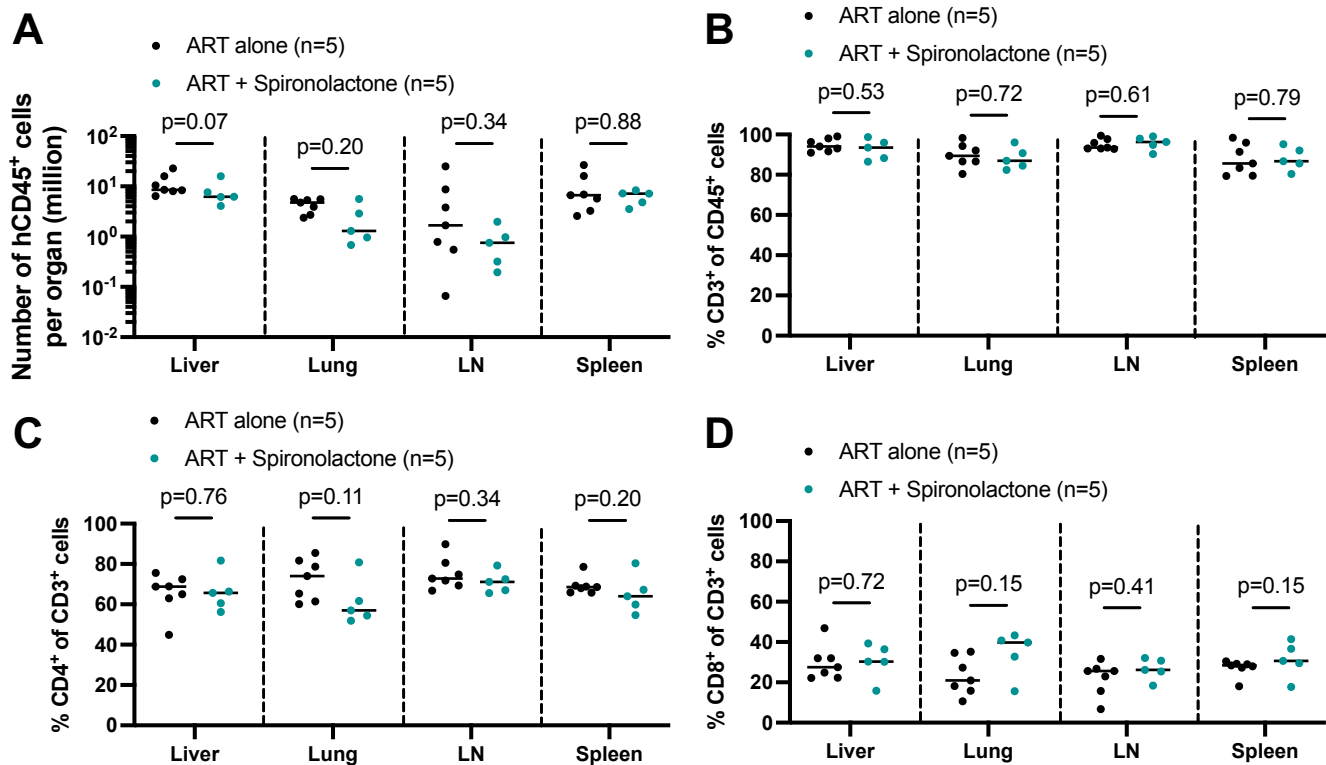

Figure S5

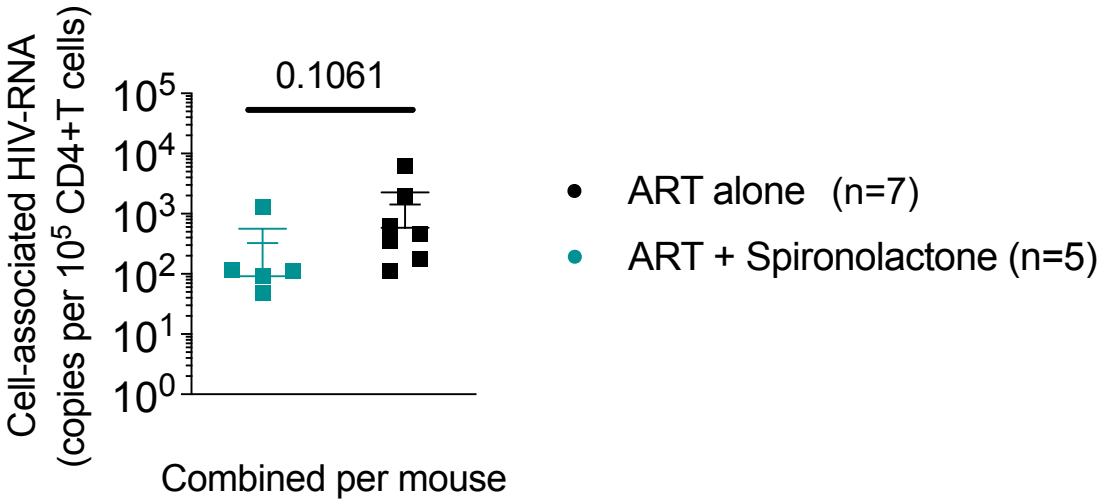

Figure S6

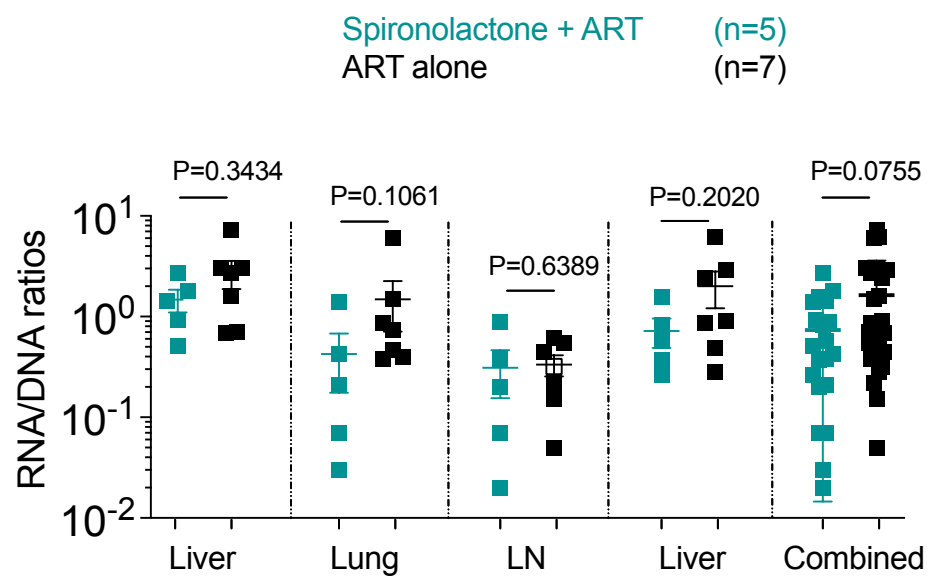

Figure S8

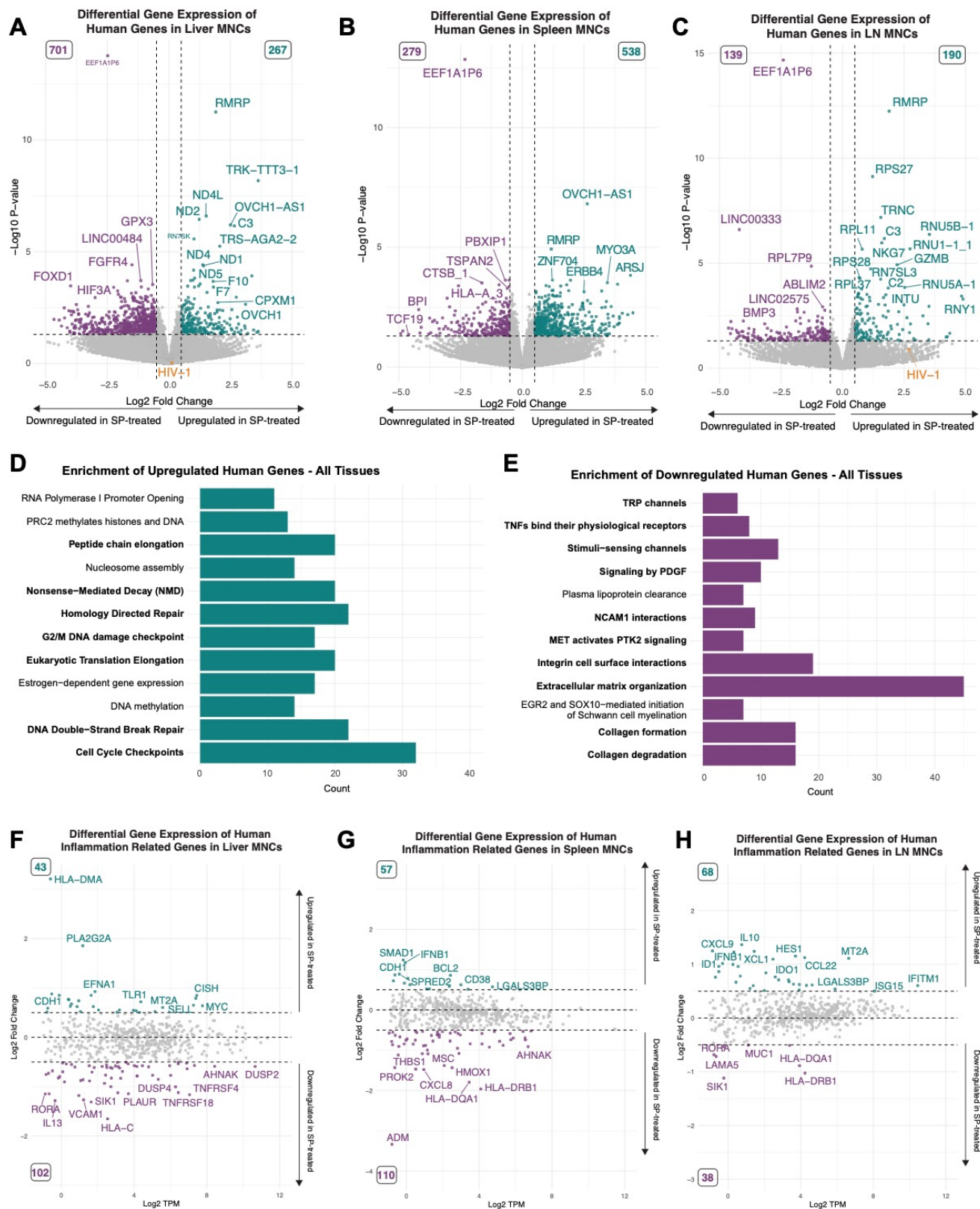

Figure S8

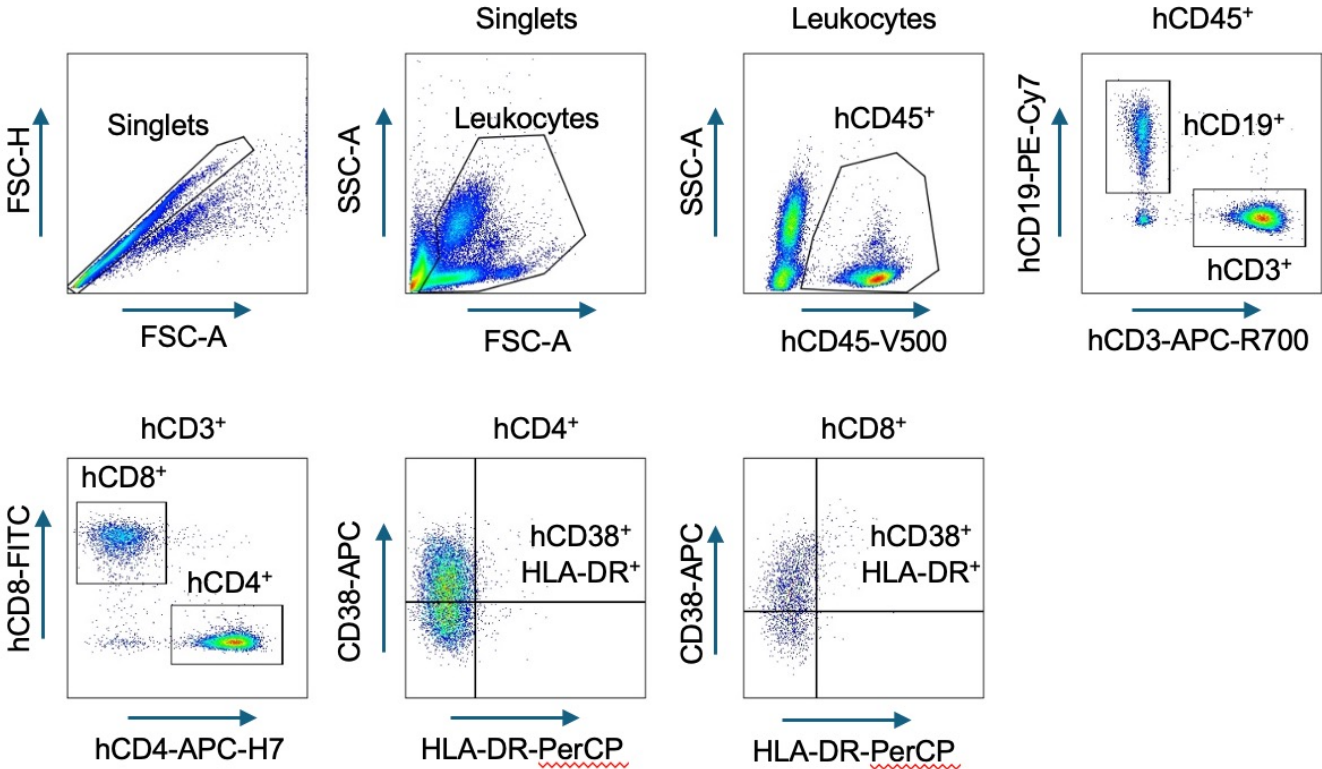

Supplement: Ling short report Supp figs.pdf [file TEMI_A_2589549_SM7754.pdf]
